# Phospholipase A2 Receptor Autoantibodies as a Novel Serological Biomarker for Autoimmune Thyroid Disease Associated Nephropathy

**DOI:** 10.3389/fimmu.2020.00837

**Published:** 2020-04-30

**Authors:** Biao Huang, Yi Zhang, Liang Wang, Qingqing Wu, Ting Li, Jue Zhang, Qiuhua Zhang, Huiming Sheng, Jiandong Bao, Zhigang Hu

**Affiliations:** ^1^College of Life Sciences and Medicine, Zhejiang Sci-Tech University, Hangzhou, China; ^2^Department of Biotechnology, Jiangsu Institute of Nuclear Medicine, Wuxi, China; ^3^Wuxi Children's Hospital, Wuxi People's Hospital Affiliated to Nanjing Medical University, Wuxi, China; ^4^Tongren Hospital, Shanghai Jiao Tong University School of Medicine, Shanghai, China; ^5^Jiangsu Jiangyuan Hospital, Wuxi, China

**Keywords:** phospholipase A2 receptor, autoantibodies, thyroid disease, autoimmune, nephropathy

## Abstract

**Aims:** To develop a highly sensitive immunoassay for PLA2R autoantibodies and study the relationship between PLA2R autoantibodies and autoimmune thyroid disease-associated nephropathy.

**Methods:** We applied a highly sensitive time-resolved fluoroimmunoassay to quantitatively detect the concentration of phospholipase A2 receptor (PLA2R) antibodies in the serum of patients with Graves' disease, Hashimoto's thyroiditis (HT), nephrotic patients with idiopathic membranous nephropathy (IMN), and normal controls. We immunohistochemically analyzed the existing PLA2R target antigen in the thyroid tissue of patients with Graves' disease and HT, as well as the nephridial tissue of nephrotic patients with IMN.

**Results:** PLA2R antibody concentrations in the serum of normal controls, patients with nodular goiter, Graves' disease, and HT, as well as patients with IMN were 1.13 ± 0.43, 1.07 ± 0.22, 2.12 ± 2.11, 8.07 ± 4.74, and 15.91 ± 19.50 mg/L, respectively. PLA2R antibody concentration in the serum and the area under the receiver operating characteristic curve in patients with HT and IMN were increased significantly. Immunohistochemistry revealed obvious staining of PLA2R in tissues from patients with HT, with a positive rate of 66.67%.

**Conclusions:** PLA2R is a potential pathogenic target antigen for HT, and the production of PLA2R antibodies may cause autoimmune thyroid disease-associated nephropathy.

## Introduction

Autoimmune thyroid disease (AITD) includes Graves' disease and Hashimoto's thyroiditis (HT). AITD has a morbidity of approximately 2% and is named as AITD-associated nephropathy when accompanied by kidney disease. Some patients with thyroid disease experience proteinuria for several months or years after confirmation of the disease (1). HT combined with membranous nephropathy was first reported in 1976 by O'Regan et al. (2); membranous nephropathy can be classified as idiopathic and secondary membranous nephropathy according to the disease etiology. Recent studies showed that idiopathic membranous nephropathy (IMN) is generally associated with production of phospholipase A2 receptor (PLA2R) antibodies, indicating that PLA2R is a major target antigen of the disease. Serum levels of PLA2R autoantibodies may be useful for the diagnosis of IMN (3–5). Reports of AITD-associated nephropathy have increased gradually over the past 40 years; however, how membranous nephropathy is caused by AITD remains poorly understood. Because PLA2R serves as the target antigen of IMN, and IMN is associated with AITD, the relationship between PLA2R in AITD should be determined. Therefore, in the present study, we used a highly sensitive time-resolved fluoroimmunoassay (TRFIA) technique to establish a quantitative method for detecting and analyzing PLA2R antibodies in the serum of patients with several common thyroid diseases and IMN. Moreover, we performed immunohistochemical analysis of pathological samples obtained from patients to investigate the clinical significance of AITD in relation to PLA2R.

## Materials and Methods

### Chemicals and Instrumentation

Goat anti-human IgG antibodies were obtained from Jackson ImmunoResearch (West Grove, PA, USA). Recombinant PLA2Rs, DAB solution, hematoxylin, were provided by the Wuxi Jiangyuan Industrial Technology and Trade Corporation (Jiangsu, China). Goat anti-PLA2R antibodies were purchased from Abcam (Cambridge, UK). A europium-labeling kit (1244-302), including N^1^-(*p*-isothiocyanatobenzyl)-diethylenetriamine-N^1^,N^2^,N^3^,N^4^-tetraacetic acid (DTTA) and enhancement solution, was purchased from Perkin Elmer (Waltham, MA, USA). Diethylenetriaminepentaacetate (DTPA), bovine serum albumin, Tris, and Triton X-100 were purchased from Sigma (St. Louis, MO, USA). Sepharose CL-6B columns were obtained from Pharmacia Co. (Uppsala, Sweden). The 96-well polystyrene microtiter plates were obtained from Nunc International (Roskilde, Denmark). All other reagents were of analytical grade and acquired from domestic manufacturers.

A model DU-650 spectrometer from Beckman (Brea, CA, USA) was used to detect proteins during antibody collection throughout the purification process. An AutoDELFIA_1235_ from Perkin Elmer was used to measure the level of Eu^3+^ fluorescence in the microtiter wells. A model AUTION MAX AX-4030 from Arkray (Edina, MN, USA) was applied as the urine automatic analyzer and a Cobas c702 autobiochemical analyzer was obtained from Roche (Basel, Switzerland). A Roche electrochemistry luminescence instrument (E170) was used to detect various thyroid serological indicators. An Olympus fluorescence microscope (Tokyo, Japan) was used for immunohistochemical analyses.

### Research Methods and Subjects

#### Preparation of Eu^3+^-Goat Anti-human IgG Antibodies

Eu^3+^-goat anti-human IgG antibodies were prepared as described previously (6). Briefly, 1 mg of goat anti-human IgG antibody was added to a small bottle containing 0.2 mg Eu^3+^-DTTA and then incubated at room temperature for 20 h. Chromatographic separation was performed on the reaction solution, and protein peaks were evaluated.

#### Preparation of the Coated Plate (6)

A volume of 100 μL of recombinant PLA2R antigen was diluted to 5 mg/L with 0.05 M carbonate at a pH of 9.6 and coated overnight. On the following day, the coating buffer was discarded, and the plate was blocked with a buffer containing 2% bovine serum albumin for 2 h. The buffer was discarded, after which the plate was evacuated under vacuum and stored at −20°C.

#### Preparation of the Standard Antibody Product

A serum from patients with IMN was added to an affinity chromatographic column connected with PLA2R recombinant antigens. The column was cleaned with TBS buffer, eluted with glycine with a pH of 2.7, monitored using an ultraviolet spectrophotometer (A280), and the first elution peak was collected to obtain a pure stock solution of the anti-PLA2R antibody. The solution was then further diluted with the reaction buffers to obtain working standard products with different concentrations.

#### Determination of the Anti-PLA2R-IgG Concentration in the Serum

The working standard product or serum (100 μL) was diluted with reaction buffer (1:100) and added to a microwell plate, which was incubated at 25°C for 1 h with agitation. After four rinses, 100 μL of Eu^3+^-goat anti-human IgG antibodies was diluted with reaction buffer and incubated at 25°C for 1 h. After six rinses, 200 μL of the enhancement solution was added followed by shaking for 5 min.

### Assessment of the Anti-PLA2R-IgG TRFIA Method

#### Sensitivity

Mean and standard deviations (SD) were calculated for count values at zero concentration points on the standard curves of 10 groups. The sensitivity was calculated as the concentration corresponding to the value of mean+2SD determined via the standard curve.

#### Precision

The intra- and inter batch coefficients of variation were determined from measurements obtained from quality-controlled samples of three different concentrations.

#### Recovery Rate

After the background response from non-specific binding was determined for the TRFIA, two anti-PLA2R-IgG standard concentrations were added to the samples. The ratio of each measured value to its theoretical value was then calculated.

#### Research Subjects

Patients from Jiangsu Jiangyuan Hospital and the Wuxi People's Hospital affiliated with Nanjing Medical University, were enrolled in the study. The study protocol was approved by the medical ethics committee of Jiangsu Jiangyuan Hospital and Affiliated Wuxi People's Hospital of Nanjing Medical University, the patients provided informed consent for the use of their samples. We assessed the thyroid color Doppler ultrasound report in combination with the patients' medical history, clinical manifestation, and laboratory examination in reference to the Diagnostic Standard for HT, Graves' disease, and Hypothyroidism in Clinical Endocrinology. There were 40 patients with HT, 47 patients with Graves' disease, and 10 patients with nodular goiter. Some patients were subjected to immunohistochemical analysis of the thyroid tissue. In addition, 46 patients were confirmed to have IMN (without cancer, lupus nephropathy, diabetic nephropathy, hepatitis B virus-associated nephritis, purpura nephritis), and 64 controls were included as healthy physical controls without thyroid disease, nephropathy, gastroduodenal disorders, or liver disease.

#### Indirect Immunofluorescence

An indirect immunofluorescence technique was applied to the thyroid surgical tissue and renal biopsy tissue. Rabbit anti-PLA2R (Sigma) was diluted (1:500) and applied to the frozen tissue. Next, horseradish peroxidase-labeled goat anti-rabbit secondary antibodies were added. The tissues were dyed using DAB solution and counterstained with hematoxylin.

### Statistical Analysis

Statistical analysis was performed using GraphPad Prism 5.0 software (GraphPad, Inc., La Jolla, CA, USA) and SPSS 19.0 software (SPSS, Inc., Chicago, IL, USA). The values are expressed as the mean ± standard deviation. Independent samples *t*-test, Wilcoxon test, and Mann-Whitney test were used to compare groups. A two-sided *P* < 0.05 was considered as statistically significant.

## Results

### Examination of Anti-PLA_2_R-IgG-TRFIA

**Figure 1:**
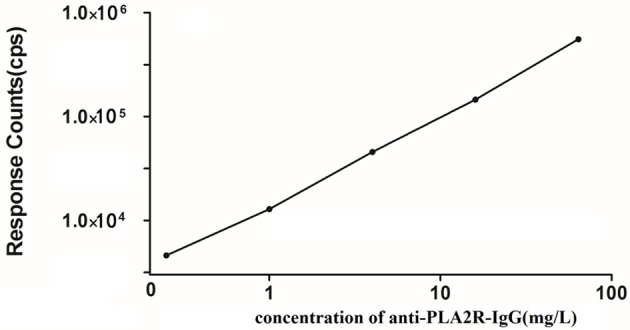
Standard curve of anti-PLA2R-IgG-TRFIA.

Figure 1 presents the anti-PLA2R-IgG-TRFIA standard curve obtained from a Log-LogB functional data processing program. The sensitivity of the method was 0.07 mg/L. The intra- and inter-batch coefficients of variation were 4.7 and 9.2%, respectively. The average recovery was 93.2%.

### Analysis of the Sample Results

**Figure 2:**
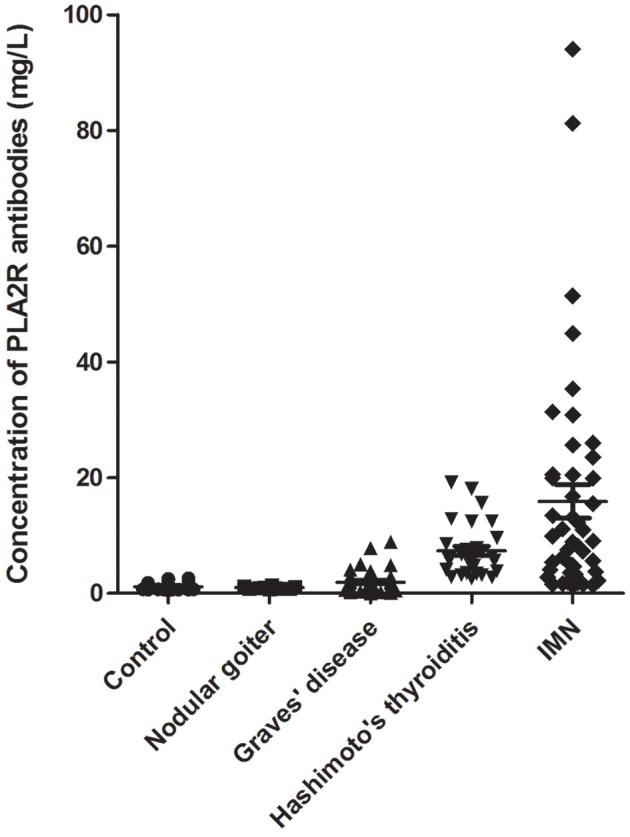
Anti-PLA2R-IgG detection in the serum of patients. Sera from 46 patients with IMN, 40 patients with HT, 47 patients with Graves' disease, 10 patients with nodular goiter, and 64 healthy controls were examined.

The samples were divided into five groups. The concentration of PLA2R antibodies in each serum sample was detected using the TRFIA method, and the results are shown in Figure 2.

Table 1 presents the level of urine protein, hematuria, serum creatinine (Scr), glomerular filtration rate, and anti-PLA2R-IgG in the serum of different patients with thyroid disease and IMN. The results showed that the positive rates of urine protein and hematuria in patients with IMN were high, and some patients tested positive for thyroid disease. The glomerular filtration rate increased in patients with graves' disease. The concentrations of serum PLA2R antibodies in patients with HT and IMN were increased significantly. The positive rate of patients with HT and IMN were 97.50%% and 82.61%, respectively.

**Table 1:** Characteristics of healthy volunteers and different patients with thyroid disease or IMN.

	**Healthy volunteers (*n* = 64)**	**Graves' disease (*n* = 47)**	**Nodular goiter (*n* = 10)**	**HT (*n* = 40)**	**IMN (*n* = 46)**
Age, years (mean ±*SD*)	39.1 ± 11.4	40.2 ± 11.4	38.2 ± 15.6	42.1 ± 13.9	44.6 ± 12.1
Male (*n*)/female (*n*)	34/30	13/34	7/3	9/31	25/21
Positive rate of Urine protein (%)	0	2.1	10	2.5	97.8
Positive rate of hematuria (%)	0	12.33	20	16.67	100
Scr (μmol/L)	63.81 ± 12.82	45.45 ± 14.17^*^	45.80 ± 13.97^*^	57.54 ± 10.27^*^	75.58 ± 35.63^*^
GFR (ml/min)	109.12 ± 11.87	126.79 ± 18.23^*^	109.12 ± 12.63	103.12 ± 16.46	101.78 ± 22.04
Anti-PLA2R IgG (mg/L)	1.13 ± 0.43	2.12 ± 2.11	1.07 ± 0.22	8.07 ± 4.74^*^	15.91 ± 19.50^*^
Range of anti-PLA2R IgG concentrations (mg/L)	0.50–2.60	0.12–8.97	0.77–1.40	2.59–21.76	1.29–94.05

*GFR (CKD-EPI) = a × (serum creatinine/b)c × (0.993) age, PLA2R, phospholipase A2 receptor; Scr, serum, creatinine; IMN, idiopathic membranous nephropathy; HT, Hashimoto's thyroiditis*.

* *P < 0.05, patient groups were compared to the healthy volunteer group*.

### Receiver Operating Characteristic (ROC) Curve Analysis

**Figure 3:**
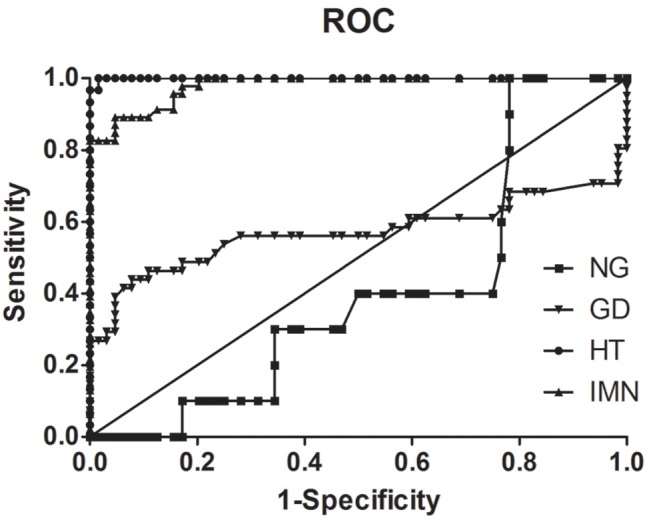
ROC curve of controls and different patients with HT, Graves' disease (GD), nodular goiter (NG), and IMN.

Figure 3 shows the ROC curve for the level of serum anti-PLA2R-IgG in controls and patients with HT, Graves' disease, nodular goiter, and IMN; the areas under the curve for the analysis of sensitivity for HT, Graves' disease, nodular goiter, and IMN were 1.000 ± 0.001, 0.569 ± 0.069, 0.402 ± 0.084, and 0.926 ± 0.027, respectively. The differences between HT, Graves' disease, or IMN and healthy volunteers were all significant (*P* < 0.01).

### Immunohistochemical Results

**Figure 4:**
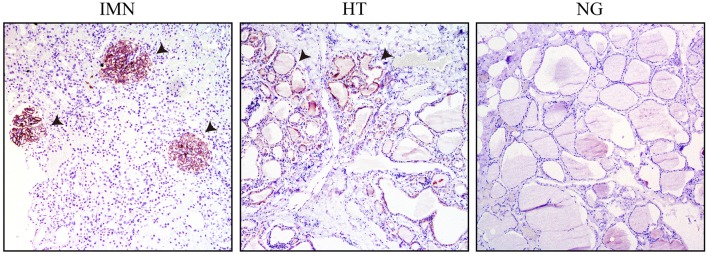
Immunohistochemical results of patients with IMN, HT, and NG, with the papillary micro-magnified by 100-fold (the arrow pointing to the positive regions indicate the glomerulus of IMN or thyroid cell members of HT).

Immunohistochemical analysis was performed on pathological sections of thyroid tissue obtained from in-patients undergoing thyroidectomy with HT and nodular goiter, as well as the nephridial tissue from patients with IMN using goat antibodies against PLA2R (Figure 4). The membrances of both sides of the thyrocytes from patients with HT and glomerular podocytes from patients with IMN were obviously stained, indicating that both patient groups contained the same PLA2R target antigens. The positive rate of tissues from patients with HT was 66.67% and tissues from patients with IMN was 84.78%; the staining in the thyroid tissues of patients with HT was weaker than that in the glomeruli of patients with IMN, indicating that the content of PLA2R in thyroid tissues was lower than that in the glomeruli of patients with IMN, and the concentration of antibodies against thyroid tissues was also lower than that of PLA2R antibodies in the serum of patients with IMN.

## Discussion

The incidence of thyroid disease and nephropathy is high, with 4.6% of the US population suffering from hypothyroidism and 1.3% from hyperthyroidism. Additionally, 10% of American adults suffer from some level of chronic kidney disease (CKD). Thyroid disorders are risk factors for CKD (7). As critical human organs, the thyroid and kidney are very closely related. Studies have shown that the thyroid can promote kidney growth and development and maintain kidney functions, and the kidney can function as a metabolic and eliminating organ for the thyroid. Therefore, AITD-associated nephropathy has attracted increased attention (8). Early in 1952, some scholars showed that 11% of patients with AITD had symptoms of proteinuria (9, 10). Patients with AITD may develop secondary renal damage, which may represent nephrotic syndrome and or less frequently renal function damage (11). Renal damage may occur prior to or simultaneously with thyroid disease symptoms. Some reports have suggested that higher thyroid-stimulating hormone levels were associated with a greater risk of subsequent CKD. Individuals with subclinical hypothyroidism and those with overt hypothyroidism are more likely to have CKD than those with euthyroid (12). The pathogenesis of AITD combined with renal damage is not completely understood and has been suggested to be associated with the formation of *in situ* immune complexes resulting from deposition of thyroglobulin and thyroid microsomal antigens in the glomerulus (13, 14). Thyroid diseases including both hypo- and hyperthyroidism are associated with several types of glomerulonephritis. The most common renal diseases observed in AITD are MN, membranoproliferative glomerulonephritis, minimal change disease, IgA nephropathy, focal segmental glomerulosclerosis, antineutrophil cytoplasmic autoantibody vasculitis, and amyloidosis. Different hypotheses have been proposed regarding the relationship between AITD and glomerulopathies, and several potential mechanisms for this association have been considered (15). The pathophysiology links between thyroid dysfunction and glomerulonephritis involve proteinuria and formation of immune complexes. This association is extremely common in autoimmune thyroiditis. These complexes are mainly responsible for alterations in renal function by depositing on the basement membrane of the glomeruli (8). However, why some renal damage occurs prior to thyroid disease symptoms is unclear. Presently, AITD-associated nephropathy is primarily diagnosed by thyroglobulin immunohistochemical staining, which shows an extremely low positive rate. Li et al. (16) conducted immunohistochemical examination of 4 patients with AITD-associated nephropathy and found that only 1 patient had thyroglobulin deposited under capillary basement membrane epithelial cells, and other 3 patients tested negative. Thus, other generic antigens may be present, and our study showed that the concentration of anti-PLA2R-IgG in the serum of patients with IMN was significantly increased. The same results were observed in patients with HT, which is also known as chronic lymphocytic thyroiditis and an AITD disease. HT, which generally occurs in middle-aged people, begins imperceptibly and slowly, and the patients would inadvertently show an enlarged thyroid while most thyroid functions remain normal; however, some patients may have symptoms accompanied by transient hyperthyreosis, typically referred to as Hashitoxicosis, and have hypothyroidism in the late stage of the disease. This disease was characterized in that high-titer antithyroid antibodies could be detected, large amount of plasmocytes and lymphocytes in thyroid tissue of patients were infiltrated to form lymphoid follicles; lymphocytes would form lymphoblasts after contact with thyroid antigens, and further produce migration inhibitory factors and lymphocytotoxins to warn the patients had T cells and corresponding antigens thereof served as thyrocyte components; it has been reported that a HT, upon initiation of treatment with levothyroxine, he had progressive deterioration in renal function and proteinuria. A renal biopsy revealed coexistent necrotizing and crescentic glomerulonephritis and membranous nephropathy. Induction treatment with oral cyclophosphamide and prednisone, at the end of 6 months of treatment, there was improvement in renal function and proteinuria (17), indicating that HT and membranous nephropath would have same pathogenesis. Anti-PLA2R-IgG has been confirmed as an autoantibody of IMN; therefore, in this study, a highly sensitive method for detecting anti-PLA2R-IgG in the serum was established with a sensitivity up to 0.07 mg/L. In a previous study, we compared in-house TRFIA and Euroimmun ELISA. Within the measurable range of the two methods, the correlation coefficient was *R*^2^ = 0.925. Because of the higher sensitivity of the TRFIA method among patients clinically diagnosed has having IMN, the ELISA positive rate was 66.7% and the in-house TRFIA positive rate was 89.7%. Thus, the results of TRFIA were better than those of ELISA (6, 18, 19). In addition, IMN can be distinguished from secondary membranous nephropathy and IgA nephropathy by setting different thresholds, which is more conducive for the differential diagnosis of IMN in the clinic (20, 21). The concentration of anti-PLA2R-IgG in the serum of patients with IMN was high, whereas its concentration in the serum of patients with HT was only slightly increased. The concentration of anti-PLA2R-IgG in the serum of patients with Graves' disease and thyroid tumor was mostly consistent with that in healthy people. Immunohistochemical analysis was further conducted on nephridial tissues from patients with IMN and thyroid tissues from patients with HT. The results showed that the PLA2R positive rate was far greater than that in thyroid tissues from patients with Graves' disease and nodular goiter patients, indicating that PLA2R is not only the target antigen of IMN but also of HT, and anti-PLA2R-IgG is not only an autoantibody of IMN, but also of HT. This may be one cause of AITD-associated nephropathy. This helps to explain why renal damage occurred before, after, or simultaneously with thyroid disease. As HT shares some symptoms with hyperthyroidism and hypothyroidism, and hypothyroidism occurs at a later stage, the generic target antigen PLA2R explains why hyperthyroidism and hypothyroidism symptoms were sometimes accompanied by nephropathy, and patients with hypothyroidism nephropathy were in a more serious condition. Therefore, PLA2R autoantibodies may be a novel serological biomarker for AITD-associated nephropathy.

## Conclusion

We developed a highly sensitive immunoassay for anti-PLA_2_R-IgG and found that the concentration of anti-PLA_2_R-IgG in the serum of patients with HT was increased. This is the first study to show that patients with HT express PLA2R antigens. Our results provide a foundation for studies of the nosogenesis and treatment of AITD-associated nephropathy.

## Data Availability Statement

All datasets generated for this study are included in the article/supplementary material.

## Ethics Statement

This study protocol was approved by the medical ethics committee of Jiangsu Jiangyuan Hospital and Affiliated Wuxi People's Hospital of Nanjing Medical University (KYL2016001). All enrolled subjects provided their written informed consent for study participation, and all methods were performed in accordance with the relevant guidelines and regulations.

## Author Contributions

BH, LW, and ZH contributed conception and design of the study. YZ, QW, TL, JZ, and JB performed the experiments. QZ, HS, and ZH performed the clinical analysis. All authors contributed to manuscript revision, read, and approved the submitted version.

## Conflict of Interest

The authors declare that the research was conducted in the absence of any commercial or financial relationships that could be construed as a potential conflict of interest.
